# Detection and Characterization of Online Substance Use Discussions Among Gamers: Qualitative Retrospective Analysis of Reddit r/StopGaming Data

**DOI:** 10.2196/58201

**Published:** 2024-10-02

**Authors:** Nicolette Le, Tiana McMann, Luning Yang, Zhuoran Li, Raphael E Cuomo, Tim K Mackey

**Affiliations:** 1 Global Health Program Department of Anthropology University of California San Diego La Jolla, CA United States; 2 Global Health Policy and Data Institute San Diego, CA United States; 3 S-3 Research San Diego, CA United States; 4 Department of Anesthesiology School of Medicine University of California San Diego San Diego, CA United States

**Keywords:** internet gaming disorder, gaming disorder, substance use, alcohol use, nicotine use, stimulants, gaming, internet gaming, video games, addiction, addiction medicine, digital mental health, reddit

## Abstract

**Background:**

Video games have rapidly become mainstream in recent decades, with over half of the US population involved in some form of digital gaming. However, concerns regarding the potential harms of excessive, disordered gaming have also risen. Internet gaming disorder (IGD) has been proposed as a tentative psychiatric disorder that requires further study by the American Psychological Association (APA) and is recognized as a behavioral addiction by the World Health Organization. Substance use among gamers has also become a concern, with caffeinated or energy drinks and prescription stimulants commonly used for performance enhancement.

**Objective:**

This study aimed to identify substance use patterns and health-related concerns among gamers among a population of Reddit users.

**Methods:**

We used the public streaming Reddit application programming interface to collect and analyze all posts from the popular subreddit, r/StopGaming. From this corpus of posts, we filtered the dataset for keywords associated with common substances that may be used to enhance gaming performance. We then applied an inductive coding approach to characterize substance use behaviors, gaming genres, and physical and mental health concerns. Potential disordered gaming behavior was also identified using the tentative IGD guidelines proposed by the APA. A chi-square test of independence was used to assess the association between gaming disorder and substance use characteristics, and multivariable logistic regression was used to analyze whether mental health discussion or the mention of any substance with sufficient sample size was significantly associated with IGD.

**Results:**

In total, 10,551 posts were collected from Reddit from June 2017 to December 2022. After filtering the dataset for substance-related keywords, 1057 were included for further analysis, of which 286 mentioned both gaming and the use of ≥1 substances. Among the 286 posts that discussed both gaming and substance use, the most mentioned substances were alcohol (n=132), cannabis (n=104), and nicotine (n=48), while the most mentioned genres were role-playing games (n=120), shooters (n=90), and multiplayer online battle arenas (n=43). Self-reported behavior that aligned with the tentative guidelines for IGD was identified in 66.8% (191/286) posts. More than half, 62.9% (180/286) of the posts, discussed a health issue, with the majority (n=144) cited mental health concerns. Common mental health concerns discussed were depression and anxiety. There was a significant association between IGD and substance use (*P*<.001; chi-square test), and there were significantly increased odds of IGD among those who self-reported substance use (odds ratio 2.29, *P*<.001) and those who discussed mental health (odds ratio 1.64, *P*<.03).

**Conclusions:**

As gaming increasingly becomes highly prevalent among various age groups and demographics, a better understanding of the interplay and convergence among disordered gaming, substance use, and negative health impacts can inform the development of interventions to mitigate risks and promote healthier gaming habits.

## Introduction

In recent decades, video games have become mainstream, with US gamers spending an average of 13 hours playing each week and over half of the US population involved in some form of digital or internet gaming [1]. Furthermore, more than 90% of children, teenagers, and young adults in the United States play video games, and these demographics spend between 4.5 and 7 hours per day [2,3]. Though the effects of video games on mental health have been examined over the years, with periodic media sensationalization bringing the discussion to the forefront of popular discussion, public debate remains ongoing over the effect of video games on individuals and broader society.

As video games have grown in popularity, concerns about the potential harmful effects of excessive and disordered gaming have also arisen and become an important topic of research. This has led to the concept of online gaming as an addiction in the 1990s and, more recently, the recognition of gaming disorder as a behavioral addiction by the World Health Organization (WHO) and the proposal of internet gaming disorder (IGD) in the latest edition of the *DSM-5* (*Diagnostic and Statistical Manual of Mental Disorders* [Fifth Edition]) as a tentative psychiatric disorder that requires further study by the American Psychological Association (APA) [2,4]. The proposed disorder has been defined as a condition of “persistent and repeated internet use for gaming, often with other players, that can lead to significant distress or impairment” and includes 9 criteria, of which 5 must be self-reported by an individual to meet the criteria for IGD [5,6]. Criteria are available in Multimedia Appendix 1. Notably, symptoms can be exhibited in either online or offline settings [3]. Since its proposal, prevalence rates of IGD have been estimated to be between 1% and 9% based on various demographics. However, among American youth, a recent study found that 8.5% of gamers met the disorder criteria [3,7].

Concerningly, mental health concerns and IGD can co-occur together and can share similar symptomology, including problems in an individual’s personal, social, and occupational life [8]. A 2018 review of the literature identified significant correlations between IGD and a number of psychological and other health problems, including bipolar disorder, anxiety, depression, and social phobia [9]. Similar findings were reported in a 2019 survey-based study, which also identified associations between problematic video game use and lower self-esteem, life satisfaction, perceived social support, self-efficacy, and academic performance [10]. Anxiety has also been identified as a risk factor for IGD [11].

IGD also shares many core features with other behavioral and addiction disorders, including substance use disorder [12,13]. Both are thought to share underlying factors, including impulsivity and escaping reality [13,14]. Hence, their intersection has become a growing area of research. Specifically, certain substances have significant use among gamers, with caffeine, energy drinks, and prescription stimulants commonly used for performance enhancement [15,16]. Recent studies have found 40% of adult gamers use performance enhancing drugs [17]. Furthermore, substance use and excessive gaming can share a bidirectional relationship with excessive gamers at an increased risk of alcohol, tobacco, and cannabis use and an earlier onset and increased consumption of certain substances correlated with increased gaming [13].

However, there remains limited research examining social media discussions of substance use and disordered gaming co-occurrence, despite that gamers commonly use online discussion forums (such as Reddit [Advance Publications], Discord, and dedicated online gaming community boards and groups) to talk to others both during online gameplay, and in offline settings. Online discussion forums represent access to important communities, which provide rich contextual information regarding gaming, as well as increased transparency among those who post due to the ability to remain anonymous [18]. Hence, this study aimed to identify substance use patterns and health-related concerns among gamers who have engaged in online discussions on Reddit regarding compulsive gaming or video game addiction, adding to the growing body of literature on video games, disordered gaming, and health. Furthermore, we aimed to assess the potential relationship of individuals who self-reported IGD characteristics, substance use co-occurrence, and health concerns among a specific Reddit gaming community and potential influences by game genre.

## Methods

### Participants

The subreddit r/StopGaming is a community on Reddit that is described as a space to “to help those who struggle with or have struggled with compulsive gaming or video game addiction,” with an estimated 52,000 members and ranked in the top 3% of communities on Reddit by size, at the time of data collection.

### Procedure

For this study, the public streaming Reddit application programming interface was used to collect and analyze all publicly available posts from r/StopGaming. Reddit was chosen based on previous studies that have used the platform to characterize substance use and gaming behaviors separately [19,20]. First, to ensure this subreddit is appropriate for examination, the dataset was filtered for keywords related to disordered gaming, including “internet gaming disorder” and “gaming addiction.” Then, to identify, characterize, and elucidate substance use behavior among habitual gamers, the dataset was filtered for keywords associated with substances commonly used to enhance gaming performance and experience (eg, stimulants, depressants, and other substances) [15]. The full list of study keywords is available in Textbox 1. An inductive coding approach was then applied to all initial posts that contained selected substance use-related keywords of interest to characterize self-reported substance use, game genres, and physical and mental health concerns. Potential disordered gaming behavior was also identified through self-reports using the tentative guidelines for IGD, as proposed by the APA. Coding criteria are available in Multimedia Appendix 1. To facilitate analysis, individual games within a series were grouped into a single property. Genre coding was determined based on the commercial market’s classification of the game [21,22]. However, each game was assigned a single genre, chosen to most comprehensively describe the gameplay. Genre categorizations included the following: role-playing games, shooters, multiplayer online battle arenas, action-adventure, simulation and sports, strategy, survival horror, platformer, puzzler and party, sandbox, and other (ie, encompassing all other gaming genre categories not fitting the genres described). First through fourth authors coded the data for study inclusion and inductively coded themes and achieved a high intercoder reliability (Cohen κ=0.95). Discrepancies in coding were discussed among authors, and a consensus on the correct classification was reached.

### Data Analysis

Mean imputation was used to provide a complete dataset for continuous variables, and mode imputation was used for categorical variables. A chi-square test of independence was used to assess the association between IGD and substance use characteristics, and multivariable logistic regression was used to analyze whether mental health discussion or the mention of any substance with sufficient sample size (ie, nicotine, caffeine, alcohol, or cannabis) was significantly associated with IGD after accounting for the association with substance use.

**Keywords related to disordered gaming and substance use were used for dataset filtering of posts collected from r/StopGaming.**

**Disordered gaming-related keywords:**

- IGD
- internet gaming disorder
- gaming addiction
- game addiction
- video game addiction

**Stimulant, depressants, and other substance-related keywords:**

- Nicotine
- Vape
- Vaping
- Cigarette
- Cig
- Adderall
- Addy
- Vyvanse
- Ritalin
- Alcohol
- Booze
- Drinking
- Energy drinks
- Soda
- Caffeine
- Weed
- Cannabis
- Smoke
- Smoking
- Drugs
- Stimulants
- Marijuana
- Cocaine
- Meth

### Ethical Considerations

This study involves the analysis of publicly available data from Reddit, specifically posts from the r/StopGaming subreddit. The data used in this research are entirely anonymous and deidentified. No personally identifiable information was collected or analyzed.

## Results

A total of 10,551 posts dated from June 2017 to December 2022 were collected from r/StopGaming. After filtering the dataset for disordered gaming-related keywords, 736 posts were identified, averaging 367 words per post, and authored by 649 unique Reddit usernames. After filtering the dataset for substance-related keywords, 1057 posts, averaging 443 words per post and authored by 937 unique usernames, were included for further analysis. Out of these, 27.1% (286/1057) posts contained self-reports of both gaming and the use of 1 or more substances. The overwhelming majority (277/286, 96.9%) were written in first person about the user themselves, while the rest (9/286, 3.1%) were written in third person about someone in the user’s personal life. The age of the individual engaged in gaming and substance use was self-reported in 30.4% (87/286) posts, with a median age of 27 (range 14-45). The summary of detected health topics, substances, and gaming genres and their frequency is available in Table 1.

**Table 1:** Qualitative characteristics of discussions found within r/StopGaming posts.

Characteristics		Count
**r/StopGaming output, n**		
	Posts collected from r/StopGaming	10,551
	Posts with relevant keywords	1057
	Discussion of gaming and substance use (signal posts)	286
**Discussion topics among signal posts (n=286)** **, n (%)**		
	Discussion of specific game genres	132 (46.2)
	Discussion of health	180 (62.9)
	Discussion of mental health	144 (50.3)
	Potential IGD^a^	191 (66.8)
**Discussion of game genre** **(n=350)** **, n (%)**		
	Role-playing game	120 (34.3)
	Shooter	90 (25.7)
	Multiplayer online battle arena	43 (12.3)
	Action adventure	27 (7.7)
	Simulation and sports	26 (7.4)
	Strategy	16 (4.6)
	Survival horror	10 (2.9)
	Platformer	6 (1.7)
	Other	6 (1.7)
	Puzzler and party	4 (1.1)
	Sandbox	2 (0.6)
	Total mentions of genres	350 (100)
**Discussion of specific substance in posts** **(n=286 posts; n=372 total mentions)** **, n (%)**		
	Alcohol	132 (46.2)
	Cannabis	104 (36.4)
	Nicotine	48 (16.8)
	Other	40 (14)
	Caffeine	31 (10.8)
	Prescription stimulants	10 (3.5)
	Illicit stimulants	3 (1)
	Hallucinogens	3 (1)
	Total mentions of substance	372 (100)

^a^IGD: internet gaming disorder.

Self-reported behavior that aligned with the tentative guidelines for IGD was identified in 66.8% (191/286) posts. More than half, 62.9% (180/286), of posts, discussed a health issue, with the majority of those posts (n=144) citing mental health concerns. Common mental health concerns discussed were depression and anxiety. Among posts that discussed both gaming and substance use, some mentioned more than 1 substance, with a total of 372 mentions of a substance in 286 posts. The majority (159/286, 55.6%) of substance use discussions indicated current use at the time of posting, while the remainder (127/286, 44.4%) indicated past use.

Discussion within the posts included a variety of topics, such as reflections on the impact of gaming disorder on authors’ lives, progress updates, and expressions of encouragement. Some authors made comparisons between their relationship with gaming and their relationship with substance use. The tone of the posts varied widely, ranging from expressions of deep struggle and continued efforts to overcome the disorder to feelings of embarrassment or shame for how disordered gaming has negatively affected areas of their lives (eg, school and academic performance, personal relationships, job performance), and also sincere appeals for support and guidance from others in the community. The selected examples are available in Table 2, which have been truncated for brevity and anonymity.

The most mentioned substances in all posts collected were alcohol (132/372, 35.5%), cannabis (104/372, 28.0%), and nicotine (48/372, 12.9%). These numbers likely underrepresent the use of cannabis and nicotine due to ambiguous discussions related to “smoking” without specifying a substance. These instances were instead coded as “Other.”

There were 133 posts that identified at least 1 specific game name, title, or game genre. Within these, there were 350 mentions of a specific name or game genre. The genres most mentioned were role-playing games (120/350, 34.3%), shooters (90/350, 25.7%), and multiplayer online battle arenas (43/350, 12.3%). Overall, 120 distinct gaming properties were identified across all posts. The top 3 properties, by number of times mentioned, accounted for over two-thirds of all mentions. These were League of Legends (37/120, 30.8%; Riot Games), World of Warcraft (25/120, 20.8%; Blizzard Entertainment), and Call of Duty (19/120, 15.8%; Activision).

The chi-square test for independence between IGD and substance use disorder revealed a statistically significant association (*χ*²_1_=29.83, *P*<.001). The expected percentage for individuals without substance use and without IGD was approximately 31.2% (330/1057), compared with the observed percentage of 36.6% (387/1057). For individuals with substance use and without IGD, the expected percentage was about 11.6% (123/1057), while the observed percentage was 6.3% (67/1057). For those without substance use and with IGD, the expected percentage was around 49.3% (521/1057), with the observed percentage being 44% (465/1057). For individuals with both substance use and IGD, the expected percentage was approximately 18.3% (193/1057), whereas the observed percentage was 23.6% (249/1057). These observed percentages significantly deviated from the expected percentages, underscoring a meaningful relationship between IGD and substance use.

In the multivariable logistic regression model where IGD was the dependent variable and substance use and discussions of mental health impacts were covariates, the model demonstrated successful convergence with a log-likelihood of –624.03 after 5 iterations. For the substance use variable, the odds ratio (OR) was 2.29 (95% CI 1.69-3.11; *z*=5.36; *P*<.001), with the odds of having IGD as 69% for those with substance use and 30% for those without substance use. For the impact on physical and mental health covariate, the OR was 1.64 (95% CI 1.05-2.56; *z*=2.17; *P*=.03), with the odds of having IGD as 62% for those with physical and mental health impacts, compared with 38% for those without such impacts. Nonsignificant associations included nicotine use (28% for individuals using nicotine compared to 29% for those not using nicotine; OR 0.96, 95% CI 0.85-1.08; *z*=–0.594; *P*=.55), caffeine use (36% for individuals not using caffeine compared with 37% for those not using caffeine; OR 0.99, 95% CI 0.89-1.11; *z*=–0.276; *P*=.78), alcohol use (40% for individuals using alcohol compared to 39% for those not using alcohol; OR 1.01, 95% CI 0.90-1.12; *z*=0.239; *P*=.81), and cannabis use (31% for individuals using cannabis compared with 32% for those not using cannabis; OR 0.98, 95% CI 0.88-1.10; *z*=–0.377; *P*=.71).

**Table 2:** Selected example posts from r/StopGaming (truncated for brevity and anonymity).

Themes	Examples
Author reflects on money spent on games and missed life opportunities.	“This year I've spent $2000 on games I don’t play. I don’t enjoy video games anymore. I hate them but I play because of habit and boredom. I'd rather have that money now. I want to sell my PlayStation and never turn back. I lost so many opportunities because I would smoke weed and play video games all day like a loser. It's time I take accountability for my actions and turn away from what makes me miserable. Instead of praying to God for a change I'm going to make a change myself. Will I have the sudden urge to play games? Yes, but I can do something else like reading or drawing. I'm sorry video games, but I'm done with you. I'm 25 now, I have shit I have to do. I ignored a lot of people who are no longer in my life because I put YOU first.”
Author compares gaming addiction to drug addiction.	“[…] Gaming is barely seen as addictive. I only recognized I was addicted after feeling the extreme emotional high that reminded me of doing a lot of cocaine. I recognized “… I have a problem”. Addiction is rooted in denial, and the very disease of addiction makes you think that you have no problem. If people aren't aware of the fact that gaming can be addictive at all, then there is no problem, right? Look at the sub count in this bar. There are 32,000 subs here. Marijuana is barely seen as addictive in mainstream culture, and yet /r/leaves has over 4 times the number of subs. […]”
Author discusses reasons for quitting and offers insights from alcohol recovery.	“[…] I'm sure I have a similar story to most of you, but I'm likely older than most (age redacted). Like many addicts, I'm chronically depressed and have a pretty negative internal dialogue. Quitting games will give me the opportunity to be more “in the moment” and spend time on activities that make me happy. IDK how often I'll check in here because, for me, Reddit and gaming are intertwined to a high degree. But I'm here to learn and offer any advice I can from my own recovery from alcohol.”
Author reflects on how time spent playing games impacting academic progress.	“Online gaming with friends can be fun, but I am depressed by the amount of my life that I wasted in a virtual world. I have over 400 hours in games. I was looking at the numbers today and became quite depressed. I have barely played any games for the past 3 months because I have been so burnt out with school. I am a year and a half behind in college. I withdrew from multiple semesters because I was so addicted to videogames. I would skip class and not do homework so that I could play games all day. Then I'd take Adderall so I wouldn't sleep and play even more. […]”

## Discussion

As video games become widely mainstream across diverse demographics of users (including console gaming, PC gaming, mobile gaming, and through immersive technologies such as virtual reality gaming), the concepts of disordered gaming and video game addiction have gained considerable attention in the public eye, leading to several countries having established protocol for regulating gaming behavior and diagnosing and treating IGD [3,23]. Previous research has found reason for concern with similarities drawn between disordered gaming and behavioral and addiction disorders, including substance use disorder, and has highlighted concerns for the implications disordered gaming has on social behavior and mental health [12,14].

This study focused on a subset of gamers who already self-identify as experiencing problematic gaming behaviors evident from their posts in a dedicated gaming addiction community, r/StopGaming, on the social networking website Reddit. In these posts, individuals report various disordered gaming behaviors and the impacts disordered gaming has had on their lives, the vast majority from a first-hand standpoint. Among the 1057 posts we analyzed, more than a quarter (286/1057, 27.1%) reported the use of at least 1 substance and active gaming. Furthermore, findings reveal that a significant portion, over 3-quarters, of those who self-report the use of at least 1 substance also meet the proposed criteria for IGD based on self-reported behavior. A smaller percentage of those who self-reported IGD-related behavior also self-reported the use of at least 1 substance. These findings suggest that substance use could contribute to the development of IGD, which supports previous research that increased consumption or earlier onset of use of certain substances may correlate to increased gaming [13].

Furthermore, role-playing, shooter, and multiplayer online battle arena games emerged as the most frequently mentioned genres. These findings are similar to those from a previous questionnaire-based study of 613 participants, which found the risk of gaming disorder to be approximately 2 times higher in individuals who preferred first-person shooter games and massively online role-playing games [2]. These observations may indicate that certain genres and games may be more prevalent among gamers who engage in problematic gaming behavior or may have features that promote addictive behaviors inherent to their design or gameplay. However, just as with the identified gaming properties, it important to note that these genre preferences could also reflect broader trends in gaming choices and may simply be representative of the distribution of popular games and genres among the general gaming population.

The study found significant associations between IGD and adverse mental health impacts, underscoring the complex and potentially harmful interplay of gaming, substance use, and mental health. These findings align with a previous review, which also found significant correlations between IGD and mental health conditions, depression, and anxiety [9]. While this study did not seek to elucidate the authors’ gender from their posts, the same review found higher video game usage and higher levels of IGD among males [9]. Previous research focused on adolescent males has found associations between playing video games and poorer social and mental health [24]. These findings highlight the importance of recognizing disordered gaming as a significant psychological concern and, potentially, an emerging public health and men’s mental health issue, though recent news reports also suggest that the number of female gamers is also increasing as a whole and requires further attention in the context of unique risk factors potentially associated with IGD [25].

Yet, it is crucial to acknowledge that this study’s findings are specific to a subset of gamers, those who see themselves as having some degree of gaming addiction due to their participation in the online r/StopGaming community. As such, it may not capture the diversity of gaming behaviors and attitudes among all gamers and lacks generalizability to the general population of all those who play video games (eg, there are also other online forums where users discuss gaming disorders, addiction, and relapse, such as the forum “Game Quitters” and its associated Discord channel). In addition, though Reddit offers users a significant degree of anonymity through features like customizable usernames, participation in topic-specific subreddits without revealing personal information, and the option to create throwaway accounts for sensitive discussions, self-reported measures remain susceptible to recall bias and social desirability bias, which could lead to over- or underreporting of behaviors. Furthermore, factors such as age, gender, and specific gaming preferences may have influenced both gaming behaviors and substance use patterns, acting as potential confounding variables. Finally, the categorization of video games by one 1 single genre that best captures overall gameplay is imperfect, as video games may be multifaceted, offer a variety of gameplay options, and have features that encompass more than 1 genre.

Future studies should focus on using validated measures to gain deeper insights into the motivation behind problematic gaming behavior and track longitudinal behavioral changes over time to explore the cross-cultural influences on gaming behaviors and substance use attitudes. A better understanding of the influence of particular games and genres, as well as online versus offline play, on disordered gaming behavior will also require a more robust and standardized framework for defining different elements of gameplay. However, the associations identified do suggest a need for the development of more accessible interventions tailored to individuals engaging in problematic gaming behaviors. As gaming continues to grow as a US $347 billion global market with widespread popularity and increasingly diverse options to play and compete (eg, esports), a better understanding of the interplay and convergence between disordered gaming, substance use, and negative health impacts can inform the development of targeted interventions aimed at mitigating risks and promoting healthier gaming habits needed for a population of multigenerational gamers worldwide [26].

## Appendix

Multimedia Appendix 1 Coding Criteria for Disordered Gaming on r/StopGaming Based on *DSM-5* (*Diagnostic and Statistical Manual of Mental Disorders [Fifth Edition]*) Proposed Criteria for Internet Gaming Disorder.

## Abbreviations

APA: American Psychological Association
*DSM-5*: *Diagnostic and Statistical Manual of Mental Disorders (Fifth Edition)*
IGD: internet gaming disorder
OR: odds ratio
WHO: World Health Organization

## Data Availability

The datasets generated during and/or analyzed during this study are available from the corresponding author on reasonable request.

## References

[1] Totilo S Number of gamers in U.S. dips slightly to 216 million.

[2] Ünal E, Gökler ME, Turan Ş (2022). An evaluation of the factors related to internet gaming disorder in young adults. Addict Health.

[3] Gentile DA, Bailey K, Bavelier D, Brockmyer JF, Cash H, Coyne SM, Doan A, Grant DS, Green CS, Griffiths M, Markle T, Petry NM, Prot S, Rae CD, Rehbein F, Rich M, Sullivan D, Woolley E, Young K (2017). Internet gaming disorder in children and adolescents. Pediatrics.

[4] Darvesh N, Radhakrishnan A, Lachance CC, Nincic V, Sharpe JP, Ghassemi M, Straus SE, Tricco AC (2020). Exploring the prevalence of gaming disorder and Internet gaming disorder: a rapid scoping review. Syst Rev.

[5] Luo T, Wei D, Guo J, Hu M, Chao X, Sun Y, Sun Q, Xiao S, Liao Y (2022). Diagnostic contribution of the DSM-5 criteria for internet gaming disorder. Front Psychiatry.

[6] (2013). Diagnostic and Statistical Manual of Mental Disorders: DSM-5. 5th ed.

[7] Gentile D (2009). Pathological video-game use among youth ages 8 to 18: a national study. Psychol Sci.

[8] Cleveland Clinic Video game addiction: What it is, symptoms & treatment.

[9] González-Bueso V, Santamaría JJ, Fernández D, Merino L, Montero E, Ribas J (2018). Association between internet gaming disorder or pathological video-game use and comorbid psychopathology: a comprehensive review. Int J Environ Res Public Health.

[10] von der Heiden JM, Braun B, Müller KW, Egloff B (2019). The association between video gaming and psychological functioning. Front Psychol.

[11] Rho MJ, Lee H, Lee T-H, Cho H, Jung DJ, Kim D-J, Choi IY (2017). Risk factors for internet gaming disorder: psychological factors and internet gaming characteristics. Int J Environ Res Public Health.

[12] Choi S-W, Kim HS, Kim G-Y, Jeon Y, Park SM, Lee J-Y, Jung HY, Sohn BK, Choi J-S, Kim D-J (2014). Similarities and differences among internet gaming disorder, gambling disorder and alcohol use disorder: a focus on impulsivity and compulsivity. J Behav Addict.

[13] Smith KL, Hummer TA, Hulvershorn LA (2015). Pathological video gaming and its relationship to substance use disorders. Curr Addict Rep.

[14] Marques LM, Uchida PM, Aguiar FO, Kadri G, Santos RIM, Barbosa SP (2023). Escaping through virtual gaming-what is the association with emotional, social, and mental health? A systematic review. Front Psychiatry.

[15] Škařupová K, Blinka L, Ťápal A (2018). Gaming under the influence: an exploratory study. J Behav Addict.

[16] Pollack CC, Kim J, Emond JA, Brand J, Gilbert-Diamond D, Masterson TD (2020). Prevalence and strategies of energy drink, soda, processed snack, candy and restaurant product marketing on the online streaming platform twitch. Public Health Nutr.

[17] Ip EJ, Urbano EPT, Caballero J, Lau WB, Clauson KA, Torn RA, Palisoc AJL, Barnett MJ (2021). The video gamer 500: performance-enhancing drug use and internet gaming disorder among adult video gamers. Comput Human Behav.

[18] Griffiths MD, Lewis AM, Ortiz De Gortari AB, Kuss DJ (2016). Online forums and solicited blogs: innovative methodologies for online gaming data collection. Stud Psychol.

[19] Chi Y, Chen H (2023). Investigating substance use via reddit: systematic scoping review. J Med Internet Res.

[20] Bergstrom K, Poor N (2021). Reddit gaming communities during times of transition. Soc Media Soc.

[21] Gameopedia (2023). Unique taxonomy in gaming: classifying video game genres.

[22] Steam Tags (Steamworks Documentation).

[23] Reuters China announces rules to reduce spending on video games.

[24] Mohammadi M, RezaeiDehaghani A, Mehrabi T, RezaeiDehaghani A (2016). Association between playing computer games and mental and social health among male adolescents in Iran in 2014. Iran J Nurs Midwifery Res.

[25] Chen V Council post: leveling up the gaming gender gap.

[26] Statista Video game industry - statistics & facts.

