# Supplementary material for: Detection and Characterization of Online Substance Use Discussions Among Gamers: Qualitative Retrospective Analysis of Reddit r/StopGaming Data
Source: JMIR Infodemiology. 2024 Oct 2;4:e58201. doi: 10.2196/58201 (PMC11483252; doi:10.2196/58201)
Supplement: Multimedia Appendix 1 [file infodemiology_v4i1e58201_app1.docx]

**Coding Criteria for Disordered Gaming on r/StopGaming Based on DSM-5 Proposed Criteria for Internet Gaming Disorder**

| Code | Explanation |
| --- | --- |
| Potential disordered gaming discussed | Post includes discussion of behavior fitting at least one proposed criterion for IGD in the DSM-V. |
| No potential disordered gaming not discussed | Post does not include discussion of behavior fitting at least one proposed criterion for IGD in the DSM-V. |

| Proposed Criteria | Description |
| --- | --- |
| Preoccupation with games | Post describes the individual consistently thinking about gaming, making it a primary focus in daily life. |
| Withdrawal symptoms | Post describes the individual experiencing symptoms such as irritability or anxiety when gaming is restricted, without physical signs of pharmacological withdrawal. |
| Increased tolerance | Post describes the individual having a growing need to invest more time in gaming activities. |
| Unsuccessful attempts to control participation | Post describes the individual making unsuccessful attempts to regulate gaming. |
| Loss of interests in previous hobbies and entertainment | Post describes the individual experiencing a loss of interest in previous hobbies and entertainment, except for those related to gaming. |
| Continued excessive use despite awareness of psychosocial problems | Post describes the individual persisting in excessive gaming despite being aware of associated psychosocial issues. |
| Deception and misrepresentation of the extent of gaming to others | Post describes the individual deceiving and misrepresenting the extent of their gaming to family, therapists, or others. |
| Escape mechanism | Post describes the individual using gaming to escape and alleviate negative moods. |
| Jeopardizing relationships and opportunities | Post describes the individual jeopardizing significant relationships, jobs, or educational/career opportunities due to excessive gaming. |
